# Experimental Investigation of a New Modular Crusher Machine Developed for Olive Oil Extraction Plants

**DOI:** 10.3390/foods11193035

**Published:** 2022-09-30

**Authors:** Antonia Tamborrino, Claudio Perone, Gianluca Veneziani, Antonio Berardi, Roberto Romaniello, Maurizio Servili, Alessandro Leone

**Affiliations:** 1 Department of Soil, Plant and Food Science (DISSPA), University of Bari Aldo Moro, Via Amendola 165/a, 70126 Bari, Italy; 2 Department of Agriculture, Food and Environment Sciences, University of Perugia, 06126 Perugia, Italy; 3 Department of Agriculture, Food, Natural Resource and Engineering, University of Foggia, 71122 Foggia, Italy

**Keywords:** crusher machine, VOO, energy, temperature, phenol content, volatile compounds

## Abstract

The crushing system is crucial in the virgin olive oil (VOO) mechanical extraction process. The use of different crusher machines can highly influence the quality of the final product, mainly due to the phenolic and volatile content responsible for VOO sensory and health properties. An experimental investigation was conducted to evaluate the effect of the geometric features of a new model of crusher machine for olives. The crusher machine consists of interchangeable rotors: a rotor with hammers and a rotor with knives. The evaluation was carried out with the same fixed grid in stainless steel with 6 mm diameter circular holes. An evaluation was carried out on the impact of the crusher tools on the pit particle size and on the distribution of energy and temperature. The performance of the plant was also assessed in terms of process efficiency and olive oil quality. The results showed that the specific energy released by the tool per unit of product, calculated through both energy conservation and comminution theory, is about 25–27% higher in the case of hammers. Since the impact energy is mainly dissipated in the product as heat, the temperature reached during milling operations with the hammer crusher was also higher by the same percentage with respect to the knife crusher. This has important consequences on the quality of the product: the new knife rotor used in the crushing phase produced an improvement in VOO quality, relating mainly to sensory attributes and the health-enhancing properties of the final product. The ability of the crusher to break cell walls and vacuoles, thus releasing the oil contained therein, is comparable for the two different rotors.

## 1. Introduction

Crushing is a mechanical operation carried out on olives after the cleaning phase. The fruit is subjected to a mechanical action that breaks the pit/stone, the walls of the vegetable cells and above all the cell vacuoles that contain oil droplets, generating a semifluid paste composed of an insoluble solid phase (fragments of pits, skins and pulp) and a liquid phase (oil-in-water emulsion) [1,2,3,4,5,6,7]. The degradation of cell walls and membranes is also achieved through depolymerization, with the activation of the enzymatic complex endogenous to the fruit (pectinase, cellulase and hemicellulase), bringing about the breakdown of cellular components and consequent leakage of cellular juices and oil, which will be more easily separable from the other constituents of the fruit in subsequent processing stages [5,8,9]. To be effective, crushing must produce (i) a fine fractionation of the pulp, obtained by applying a very small force in numerous breaking actions, and (ii) a non-excessive splitting of the woody core by applying a very large force in a few areas [4]. From a physical point of view, olive crushing entails the application of normal effort, shear stress and friction. The friction and shear stress produce the efficient disarticulation and laceration of plant tissues, with low energy dispersion, causing a low heating of the mass [10,11]. These actions are ideal for crushing olive pulp. The normal effort required to break the kernels generates shocks, percussion, crushing and violent impacts, with high energy dispersion and significant heating of the mass. The intensity of the forces and how they act on the olive is a function of the geometry of the machine and the speed of the rotating parts of the mechanical crusher [12,13]. The crushing operation is crucial in determining the extraction yield, the quality and composition of the final product and the bioactivity level of the virgin olive oil produced [11,14]. Throughout this phase, all the endogenous enzymes of the olive fruit are activated and involved in subsequent phases in the extraction process, with them also being involved in the formation and modification of the phenolic and volatile compounds of the extra virgin olive oil (EVOO) [15,16,17]. The different mechanical aspects of crushers used in normal olive mills can result in different crushing effects, leading to different final concentrations in terms of phenolic and volatile compounds, which have significant effects on the taste, flavor and stability of the olive oil and thus the quality of the EVOO [18,19,20,21]. Currently, there are different models of olive crushers available for industrial operations, such as the traditional stone mill, the hammer crusher, the disc crusher, the so-called toothed crusher, and destoning machines. Each crusher applies a different and specific mechanical action to break down the olive tissue, creating several effects [4]. These effects play an important role in endogenous enzymatic activities, affecting the final amount of EVOO as well as the phenolic and volatile profiles [20,21]. Some studies on the crushing phase have investigated the effects of different crushers on the composition and overall quality of olive oil. In 2003, Caponio et al. [18] found that the hammer mill makes it possible to extract greater amounts of phenols from plant tissues than stone mills, and consequently the oil produced with a hammer crusher is more bitter, with a greater antioxidant capacity, than that obtained with stone mills. This was confirmed by Inarejos-García et al. [19]. The diameter of the holes and speed of rotation are also technological parameters to be considered during the crushing process, as they determine the thickness of the paste and thus the composition and quality of the final EVOO produced, as investigated by Inarejos-García et al. [19]. These results are in accordance with those reported by other authors [14,17,21,22]. The present study entailed a set of experimental tests on a new machine, a new type of modular mechanical crusher developed in partnership with a private company manufacturing machines for the extraction of olive oil. The new crusher machine model was developed with an interchangeable rotor to adapt to the characteristics of the olives and to the type of oil one wishes to obtain. The fitted rotors were a rotor with hammers and a rotor with knives. In this way, it was possible to separately evaluate the influence of the actions induced by the crushing organs of the rotors, leaving all the other construction and process parameters unchanged. The numerous effects of the crushing system observed on the quality of olive oil are due to the interaction of the pressure and shear imparted by the machine on the olive fruits. Thus, one of the aims of the present study was to investigate the effect of the geometric features on the crushing action by means of a simplified model for the energy transfer and dissipation of the crushers. Analysis of the specific energy produced by the crusher, combined with an investigation into the particle size fraction after crushing, should reveal the prevailing mode of action of forces on the fruit. The study also analyzed the average energy per unit of mass for both rotors in order to understand how the mechanical parts affect the distribution of forces on the olives. Finally, the research also sought to evaluate the effects on the yield and quality of the oil obtained using the two different processing systems.

## 2. Materials and Methods

### 2.1. The Modular Crusher Machine

**Figure 1:**
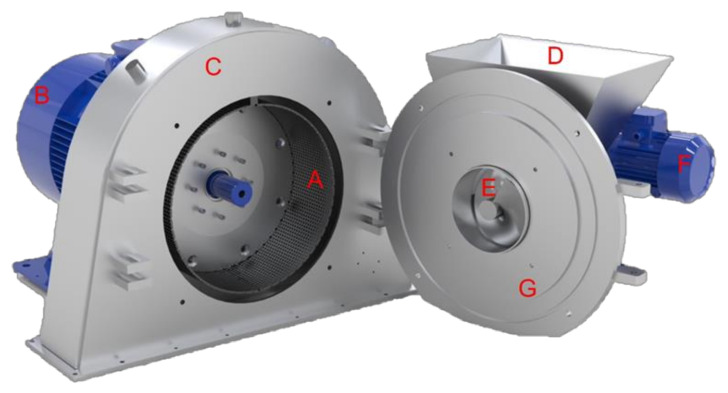
Crusher machine: (A) grid, (B) electric motor for rotor, (C) crankcase, (D) loading hopper, (E) feeding screw, (F) electric motor for feeding screw, (G) inspection hatch.

**Figure 2:**
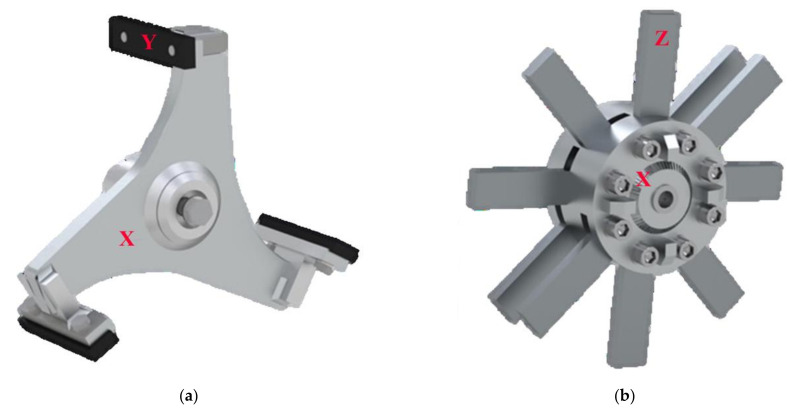
(**a**) Hammer Crusher Rotor: (X) rotor, (Y) hammer. (**b**) Knife Crusher Rotor: (X) rotor, (Z) knife.

The modular olive crusher machine was developed using a standard crusher base frame, as shown in Figure 1, equipped with two different types of rotors for crushing olives: a rotor in which the classic hammers, commonly used for crushing, were mounted and a new type of rotor in which, instead of hammers, knives are mounted and placed radially in relation to the rotor shaft (Figure 2a,b). The crusher was sized to work with an olive mass flow rate of up to 15 t h^−1^. The basic structure consists of:

- a fixed stainless steel grid (with a thickness of 5 mm, depth of 182 mm and diameter of 460 mm) with circular holes, each having a diameter of 6 mm;
- a main electric motor (two-poles, 30 kW, 3000 rpm);
- an external carter to collect the olive paste coming out of the grid, open in the lower part;
- an integrated closing door, screw and hopper system to feed the incoming olives;
- a secondary electric motor (two-poles, 30 kW) and reducer, connected to the screw feeding the olives.

The hammer rotor was built with three spokes, at the end of which the hammers were mounted on spacer guides. The hammer is a parallelepiped-shaped tool with a size of 5 mm × 25 mm × 155 mm (cutting side), made of mild steel, which is mounted at the end of each spoke on a guide, making it possible to vary the distance from the grid from 5 mm to 10 mm.

The knife rotor consists of five overlapping fringes with a diameter of 180 mm containing 10 radial knives made of mild steel. The knives are positioned in three rows and are offset by 45° from each other. Two pairs of knives are double. Each knife has a cut length of 117 mm and is positioned 18 mm from the grid.

### 2.2. Experimental Design

**Figure 3:**
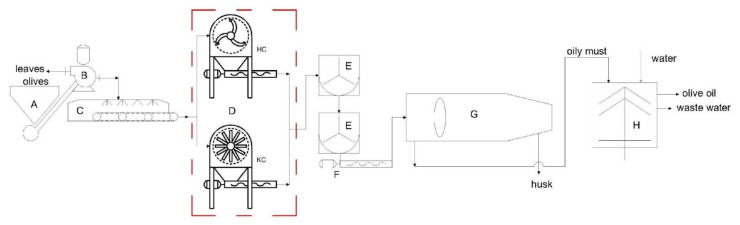
Layout of industrial olive oil extraction plant and process: (A) loading hopper; (B) defoliator; (C) washing machine; (D) crusher machines; (E) malaxer machines; (F) cavity pump stators; (G) solid/liquid horizontal centrifugal decanter; (H) liquid/liquid vertical centrifuges.

Crushing tests were carried out using the crusher machine equipped with the interchangeable rotor: a hammer crusher (HC) and knife crusher (KC). The machine was installed in two different industrial oil extraction lines, one at the Guglielmi Oil mill in Andria (BAT) and the other at the Produttori Olivicoli Bitonto mill in Bitonto (BA), Italy. An experimental oil extraction design was carried out in both mills using the crusher machine and alternating between the two experimental rotors. In all tests, the same external grid and the same base frame of crusher machine were used, with only the rotor type being exchanged. The two extraction lines used in the two different oil mills were similar, and both were installed by Amenduni Nicola s.p.a. (Bari). In greater detail, the line at the Guglielmi Oil mill consisted of: (i) the olive cleaning unit (mod. Clemente Ocean 1); (ii) a modular crusher; (iii) a piston pump (mod. PP10 single effect); (iv) two malaxers connected in series (mod. V6000); (v) cavity pump stators (mod. Bellin 3000); (vi) a 2-phase decanter (mod. REX250); and (vii) a separator (mod. A3500). At the Produttori Olivicoli Bitonto mill, the line consisted of: (i) the olive cleaning unit (mod. Clemente Ocean 1); (ii) a modular crusher; (iii) a piston pump (mod. PP10 single effect); (iv) two malaxers connected in parallel (mod. 6V1000); (v) cavity pump stators (mod. Bellin 3000); (vi) a 2-phase decanter (mod. REX250); and (vii) a separator (mod. A3500). Figure 3 shows the olive oil extraction plant in the two different layouts with the hammer rotor (HR condition) and knife rotor (KC condition).

The tests were carried out at the Guglielmi oil mill in November 2020 using olive fruits (*Olea europaea* L.) from Coratina cultivar with a maturity index of 1.6, while at the Produttori Olivicoli Bitonto mill the tests were conducted in December 2021 using olive fruits (*Olea europaea* L.) from Coratina cultivar with a maturity index of 2.3. The maturity index was measured as in Beltran et al. [23].

Each test for both years was repeated 10 times using homogeneous 3000 kg batches of olives at the Guglielmi Mill and 1000 kg batches at the Produttori Olivicoli Bitonto mill. During all tests, malaxation was performed for 30 min at 24 ± 1 °C, with a plant mass flow rate of 5 t h^−1^, without the addition of dilution water. During each test, the incoming olives, olive paste, pomace leaving the decanter and final oil were sampled in order to determine the extractability, olive oil lost in the pomace and quality parameters of the olive oil. Finally, particle size distribution was analyzed on the olive paste samples.

### 2.3. Particle Size Measurement

For each test, performed at Guglielmi Mill from Coratina cultivar with a maturity index of 1.6, samples of olive paste were collected downstream of each crusher configuration (HC and KC), each having a mass of 10 kg. Each sample of olive paste was washed with water in order to eliminate the liquid parts and light solids (skin and pulp) and recover all the pit fragments. Subsequently, each of the 10 samples of pit fragments was sifted using a set of 16 sieves with grid diameters ranging from 0.80–5.00 mm. Thus, 17 pit size classes were obtained and weighed using analytical scales. For HC and KC configuration, the weight of the aliquots of the pit deriving from each size class were averaged. Weight size class trends were evaluated using a smoothing spline function to visualize the actual behavior of the pit dimensions. The smoothing spline function applied considered the minimum distance of the function f from the given data, measured as:

(1) $$E\left(f\right)=\overset{n}{\underset{j=1}{\sum }}w\left(j\right){\left|y\left(obs,j\right)-f\left(x\left(j\right)\right)\right|}^{2}$$

where *j* = cardinal number of data point (*x*), *n* = total number of data points (*x*), *obs* = number of observations and *w* = weight factor. The *w* value was calculated as:

(2) $$w=\int_{\min\left(x\right)}^{\max\left(x\right)}{\left|y-f\right|}^{2}$$

The mean, mode, median, sigma, kurtosis and skewness were determined for each distribution. The kurtosis (*k*) and skewness (*γ*) indexes were calculated as follows:

(3) $$k_{1}=\frac{\frac{1}{n}\sum_{i=1}^{n}{\left(x_{i}-\bar{x}\right)}^{4}}{{\left[\frac{1}{n}\sum_{i=1}^{n}{\left(x_{i}-\bar{x}\right)}^{2}\right]}^{2}}$$

(4) $$\gamma_{1}=\frac{1}{\sigma^{3}}\left[\frac{\sum_{i}{\left(x_{i}-\mu \right)}^{3}f_{i}}{\sum_{i}f_{i}}\right]$$

where *μ* represents the mean of the distribution, *n* is the number of observations, *σ* is the standard deviation and *f_i_* is the frequency associated with each *x_i_*. Kurtosis and skewness, describes the shape of a probability distribution. The distribution curves were obtained using the MATLAB^®^ Statistical and Machine Learning Toolbox (Mathworks Inc., Natik, MA, USA). The number of classes to consider was determined by Doane’s rule [24].

### 2.4. Impact Estimation by Energy Conservation and Comminution Theory

**Figure 4:**
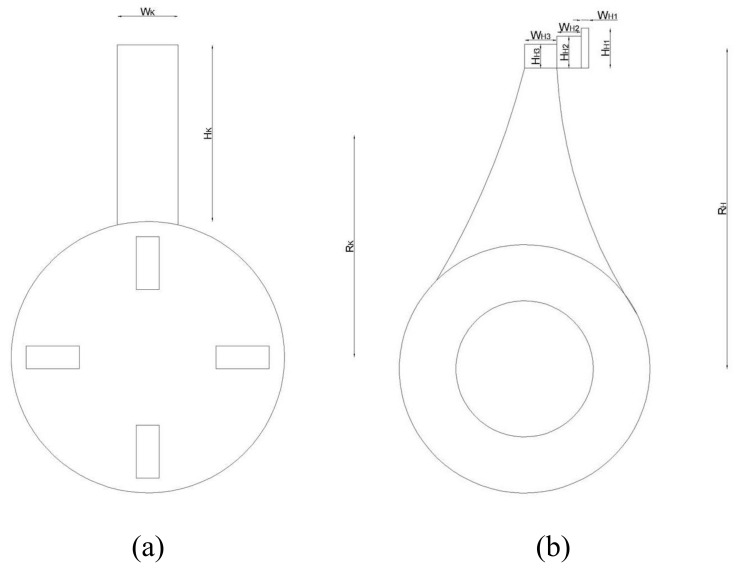
Schematic representation of a single tool of a hammer crusher (**a**) and knife crusher (**b**).

To assess the mechanical behavior of the two rotors, a simplified method based on the evaluation of the average specific impact of energy was adopted. The energy per unit of mass was estimated with the assumption of rotational kinetic energy conservation. This approach is based on the work proposed by Nikolov [25] and implemented in the study by Mugabi et al. [26]. Although this method is a simplified schematization of the crushing process, it represents an important aid to explaining some of the experimental observations made in this study. The crushing effect depends on the kinetic energy of crusher tool. In particular, it depends on the interchange of energy between the tool and olive (the loss of energy due to impact). The impact energy is strictly related to the nature and magnitude of forces, with a great effect on energy transfer and dissipation as heat inside the product. In order to compare the energy transferred (and thus dissipated in the olive paste) for the two rotors under review, a schematic representation of a single tool is shown in Figure 4: hammer (a) and knife (b). A three-dimensional view of the tools is shown in Figure 2. The conservation of kinetic energy can be estimated by neglecting the contribution of the olives’ kinetic energy, since their mass is much smaller than that of the tool:

(5) $$E_{c}=\frac{1}{2}I_{c}\omega^{2}$$

where *I* is the moment of inertia of the tool and *ω* its angular velocity (*c* is equal to H for hammer and K for knife, see Figure 4). It is worth remembering that the moment of inertia is equivalent to the mass in rotational motion. The entire mass of each tool can be assumed to be concentrated in its center of gravity, and the moment of inertia can be calculated using the well-known Huygens–Steiner theorem (the parallel axis theorem):

(6) $$I_{c}=\left(\frac{\sum \left(H_{ci}^{2}+W_{ci}^{2}\right)}{12}\right)\cdot M_{i}+R_{c}^{2}\cdot M_{i}$$

where *H* and *W* are the height and width of each tool component, *R* is the distance of the center of gravity and *M_i_* the mass of the tool.

The rotor tool impacts a certain mass of olives *M_oc_*, which can be determined as follows:

(7) $$M_{oc}=M_{o}\frac{H_{c}}{d_{o}}\frac{D_{c}}{d_{o}}$$

where *M_o_* is the mass of a single olive, *d_o_* its mean diameter, and *D* is the depth of the tool. The amount of impacting olives was calculated considering the uniform distribution on the tool. In fact, the term *H_c_*/*d_o_* represents the number of olives along the vertical direction of the tool, while *W_c_*/*d_o_* represents the number in the transversal one.

Finally, the specific energy for both crushers can be evaluated using the ratio of rotational kinetic energy to the mass of affected olives:

(8) $$e_{c}=\frac{E_{c}}{M_{oc}}$$

Another way to evaluate the energy transferred by the crusher tool is through size reduction theory (comminution). As reported in Caponio and Catalano (2001), in the case of olives we can use the Bond equation:

(9) $$e_{c}=K_{B}\left[{\left(\frac{D_{r}}{D_{2}}\right)}^{0.5}-{\left(\frac{D_{r}}{D_{1}}\right)}^{0.5}\right]$$

where *D_r_* is the reference diameter, *D*_1_ is the initial diameter of the pit, *D*_2_ is the final diameter of the pit (mean value), and *K_B_* is an empirical constant related to the resistance offered by the product. However, since the diameter of the pit is much higher than that referenced, the second term of Equation (10) can be neglected. The energy transferred to the olives, in addition to that needed to overcome friction in the moving components of the crusher, is mostly released as heat in the milled product. This leads to an increase in the temperature of the olive paste and the consequent degradation of the quality of the final product. In particular, the energy stored in the particle as internal stress is released when the particle fractures. Recalling the heat flux through the external surface of the crusher reported in Caponio and Catalano [27], the mean temperature of olive paste *T_mc_* can be evaluated as follows:

(10) $$T_{mc}=T_{e}+\frac{M_{p}}{M_{o}}\frac{e_{c}Q_{o}}{hS}$$

where *T_e_* is the environmental temperature, *M_p_* is the mean value of the pit mass, *h* is the heat transfer coefficient, *S* is the surface of the crusher through which the heat is dissipated, and *Q_o_* is the average flow rate through the crusher. It is easy to see that the temperature reached by the olive paste is directly proportional to the energy transferred to it.

### 2.5. Moisture and Oil Content of Olives and Pomace

The moisture content (% *w*/*w*) was calculated after drying milled olives and pomace from the decanter at 105 °C to a constant weight. The total oil content of dried milled olives and pomace was determined using the analytical method reported in Caponio et al. [28].

### 2.6. Extractability

The extractability parameters of the oil present in pomace and wastewater were used to evaluate the quantitative performance of the oil extraction plant. Oil extractability (E) is the ratio of the percentage of oil extracted from the olives (Oe) by the plant to the percentage of oil content in the olives (Oo). E was calculated using the following equation:

E = Oe/Oo ∗ 100(11)

### 2.7. VOO Chemical Analysis

#### 2.7.1. Solvents and Reference Compounds

Glacial acetic acid and methyl alcohol (HPLC grade) were supplied by VWR International S.r.l. (Milan, Italy). HPLC grade water was achieved using the water purifier system PURELAB ultra analytic (ELGA, Lane End, UK). Phenolic alcohols, hydroxytyrosol (3,4-DHPEA) and tyrosol (*p*-HPEA), were purchased from Cabru s.a.s. (Arcore, Milan, Italy). Pinoresinol and vanillic acid were purchased from PhytoLab GmbH and Co., KG (Vestenbergsgreuth, Germany) and Merck (Milan, Italy), respectively. The dialdehydic forms of elenolic acid linked to 3,4-DHPEA (3,4-DHPEA-EDA) and *p*-HPEA (*p*-HPEA-EDA), the isomer of oleuropein aglycon (3,4-DHPEA-EA) and (+)-1-acetoxypinoresinol were extracted from virgin olive oil as described by Selvaggini et al. [29]. Analytical standards of the volatile compounds were purchased from Merck (Milan, Italy).

#### 2.7.2. Legal Quality Parameters

The data on free acidity, peroxide value and UV spectrophotometric indices (K232, K270 and ΔK) were obtained following the methods described by Regulation (EU) 2019/1604 [30].

#### 2.7.3. Phenolic Compounds

The concentrations of 3,4-DHPEA-EDA (oleacein), *p*-HPEA-EDA (oleocanthal), 3,4-DHPEAEA (isomer of the oleuropein aglycon), *p*-HPEA-EA (ligstroside aglycon), 3,4-DHPEA (hydroxy-tyrosol), *p*-HPEA (tyrosol), vanillic acid, (+)-1-acetoxypinoresinol and (+)-pino-resinol were extracted, evaluated and quantified using an Agilent Technologies system Mod. 1100, composed of a vacuum degasser, a quaternary pump, an autosampler, a thermostated column compartment and detectors (DAD and FLD) equipped with a C18 column (Spherisorb ODS-1 (250 mm × 4.6 mm), with a particle size of 5 μm, supplied by Phase Separation Ltd. (Deeside, UK), following the high-performance liquid chromatography (HPLC) analysis described by Taticchi et al. [31]. The results were expressed in mg of phenolic compounds per kg of oil.

#### 2.7.4. Volatile Compounds

The qualitative and quantitative evaluations of the volatile compounds in VOOs were carried out by headspace-solid phase microextraction (HS-SPME) coupled with gas chromatography-mass spectrometry (HS-SPME-GC/MS) according to Taticchi et al. [32]. The volatile compounds were identified by comparing their mass spectra and retention times with those of authentic reference compounds and with the spectra in the NIST 2014 mass spectral library. Volatile compounds were quantified using calibration curves for each compound by internal standard calculation, and the results were expressed in μg/kg of oil.

#### 2.7.5. Data Processing

The MATLAB^®^ machine learning and statistical toolbox was used to process the experimental data. The significance among means of groups of data was detected using the two-tailed *t*-test hypothesis test (*p* < 0.05).

## 3. Results and Discussion

### 3.1. Pit Particle Size Distribution

**Figure 5:**
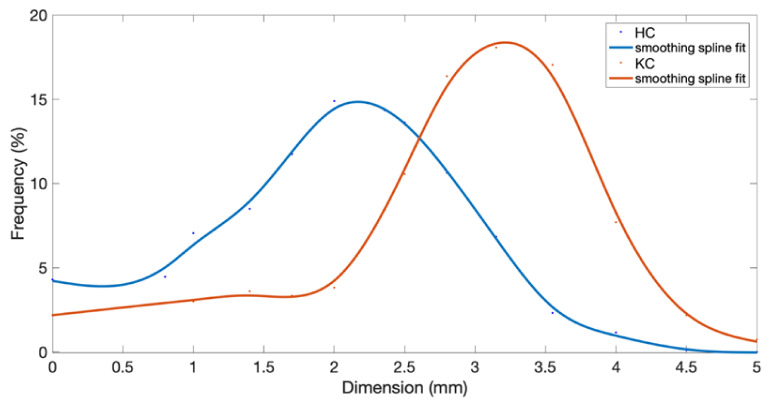
Trends of pit particle size as smoothing spline curves, for hammer crusher (HC) and knife crusher (KC).

Figure 5 shows the size frequency curves of the kernel particles obtained from the pulps crushed with the HC (blue line) and KC (red line) systems. An examination of the trends shows that the KC system produced kernel particles with a high frequency in the 3–3.5 mm size class, unlike the HC system, where the maximum frequency was in the 2–2.5 mm size class. Moreover, the KC system generated a constant, low frequency of kernel fractions below 2 mm. On the contrary, the HC system showed a production of pit particles with a high frequency in the lower size classes, compared to the higher ones, resulting in a high dimensional variability in terms of obtained kernel pieces. These substantial differences can be attributed to the impact energy of the two different systems, as described in Section 3.2.

Mean particle size produced by the HC crusher (2.00 mm ± 0.05) is significantly lower than that of the KC crusher (3.15 mm ± 0.06), as well as for the values of median, modal and sigma. Both distribution curves show moderate negative asymmetry with the tail on the left side of the distribution (−0.1 for HC distribution and −0.5 for KC distribution).

The kurtosis value is −0.4 for HC distribution curve and 0.3 for KC distribution curve. This indicates that the distribution curve of the pit fragments is platykurtic for HC, with frequencies better distributed between the classes and leptokurtic for KC, where a higher frequency concentration around the modal value is highlighted. This means that KC generate a higher percentage of large fragments than the HC.

### 3.2. Average Energy Impact and Temperature Comparison

To compare the energy transferred by the crusher tool to the olives, both energy conservation and comminution theory were used. Table 1 gives the values measured on the single tool of both crushers, referring to Figure 4.

As for the methodology based on energy conservation, we must refer to equations 5 to 8. On the basis of the values reported in Table 1, it was possible to compare the two rotor crushers, as reported in Table 2.

As Table 2 shows, the total mass of the hammer was double that of the knife. Although the hammer itself is only 5 mm in depth, the back supports contribute to the mass of the tool and have a great effect in transferring energy to the olives. The mass of the tools was calculated by their geometric dimensions (volume) and the density of the steel (considered to be the same for both hammer and knife). In addition, considering that the moment of inertia has a quadratic dependence on the distance of the center of gravity from the center of rotation (Equation (6)), since the radius of the hammer is 36% larger than that of the knife, its moment of inertia is much higher, being 3.50 times that of the knife. As a result, the rotational kinetic energy has the same relationship since the angular velocity of the rotor is the same in both cases. However, with the depth of the hammer being 155 mm and that of the knife only 12 mm, the frontal area of the former is much greater than that of the latter (although its height is greater). This means that the hammer tool affects a higher mass of olives, 2.76 times more than that of the knife. Therefore, the specific energy (Equation (8)) per unit of mass of olives (1 kg) was 27% higher in the case of the hammer crusher. The same result is achieved via the comminution theory and particle size distribution conducted in this study. As shown in Figure 5, the mean final diameter of pits with the hammer crusher was about 2.00 mm, compared with about 3.15 mm with the knife crusher. According to Equation (9), the specific energy transferred to the olives by the hammer is about 25–26% higher than that of the knife. As explained above, the impact energy is mainly dissipated in the product in the form of heat, leading to an increase in the olive paste temperature. It is easy to imagine that a higher product temperature leads to product degradation, especially the more sensitive components such as polyphenols and volatile components. Considering that the body of the crusher is the same (the same equipment to which the rotor is attached) and thus the surface and its thermal properties remain unchanged, and that the feed flow rate is the same in both cases, the mean temperature reached by the olive paste depends only on specific energy. As a result, the temperature reached during the milling operation with the hammer crusher is about 25–27% higher than that with the knife crusher. These results are summarized in Table 3.

Although these analyses are a simplification of the actual crushing process, they allow us to understand and hypothesize the prevailing modes of action of forces on the olives and explain the qualitative aspects observed in the final product. It is well known that the main forces applied during crushing are compression, impact, shear, or, more likely, a combination of these forces. It is also well known that compression and impulsive forces produce a much higher dissipation of energy stored in the product, while shear forces result in efficient size reduction with a lower dissipation of energy and, thus, less overheating with regards to the product. Therefore, it is reasonable to believe that the hammer crusher produces mainly impulsive and compressive stresses. On the contrary, the knife crusher transfers mainly shear stress. This is probably due to the shape of the tool itself: the hammer is flat and wide, while the knife is long and narrow. Consequently, the probability of the fruit being hit on the edge with the knife is much higher, and thus a shear stress is generated on the product that is trapped between the tool and the adjacent product. As for the hammer, the greater likelihood of hitting the product in the normal direction of the tool mainly produces impulsive and compressive stresses, with much higher levels of heat generation. This phenomenon has significant consequences on the quality of the product, as detailed in the following sections.

### 3.3. Quantitative Evaluation

Table 4 shows the quantitative data obtained with the two crushing systems compared in the two different harvest years with olives having two different maturity indices. As shown by the data, regardless of the maturity index of the olives, there are no significant variations as regards the oil lost in the pomace and the extractability of the final olive oil. This confirms that the ability of the crusher to break the cell walls and the vacuoles, thus releasing the oil contained therein, is comparable using the two tested rotors. This means that although the crusher in the knife configuration generates a combination of forces transferred to the olives that is less energetic than the hammer configuration, the hammer is sufficient to produce satisfactory yield results.

### 3.4. Qualitative Evaluation

The use of two different crushers on Coratina olives with two maturity indices did not affect the values of the main legal quality parameters for VOOs. The data on the free acidity, peroxide value and UV spectrophotometric indices (K232, K270 and ΔK) did not show any significant differences, with the parameters being well below the limits for extra virgin olive oil (Table 5). On the contrary, the phenolic composition was highly influenced by the application of different crushers, with a significant increase for VOOs extracted using the knife crusher. This result also appears to be influenced by the maturity indices of the olive drupes, with a larger improvement in the VOO phenolic fraction (Table 6), probably due to a larger amount of phenolic compounds contained in the olive pulp characterized by an early maturity index [33,34]. The phenolic concentration increased in KC VOOs by 34.3% and 15.6% compared with HC VOOs, respectively, for olives having a 1.6 and 2.3 maturity index. These large increases, pushing the total phenols well over 1000 mg kg^−1^, were mainly due to the concentration of oleuropein derivatives (3,4-DHPEA-EDA and 3,4-DHPEA-EA), even though the VOOs extracted from olive drupes having a lower maturity index also showed a significant improvement in oleocanthal content. The granulometric analysis of the olive paste obtained using the hammer crusher showed a lower pit particle size distribution than that obtained using the the knife crusher, with a consequent higher degradation of olive seed tissues. The olive seed contains a high level of peroxidase (POX) activity in the whole drupe [35,36] that influences the oxidative reactions of phenols, playing an important role in determining VOO phenolic content [37]. A higher granulometric fraction of KC olive paste entailed a lower level of POX activity during the mechanical extraction process, justifying the higher total phenol content of KC VOOs compared to HC VOO samples due to reduced oxidoreductase activity. The limited activity of POX, due to a mild disruption of the seed tissues or the use of a destoning machine, confirms results on the improvement of the phenolic compounds of VOOs during the mechanical extraction process [17,37,38,39,40,41]. The new knife rotor, fitted with five overlapping fringes, was able to combine the optimal rupture of the olive pulp necessary for the biogeneration of phenolic compounds [19,20,21,22,23,24,25,26,27,28,29,30,31,32,33,34,35,36,37,38,39,40,41,42], limiting the negative effects due to the oxidation process induced by POX (Luaces et al., 2007; Garcia-Rodriguez et al., 2015) [35,37]. The new knife crusher also showed a significant effect on the volatile fraction of VOOs, probably due to energy transferred to the olive paste, improving lipoxygenase (LOX) activity. Based on data from previous studies that showed an enhancement that ranged from 5% to 33% due to different cultivars [14,18,41,43], Table 7 shows an increase in the sum of aldehydes for both olive batches having a different maturity index. The increases were mainly due to an enhancement of the content of (E)-2-Hexenal improving the green and cut-grass sensory notes of the final product. The lower temperature of the olive paste during the crushing phase, due to the use of knives, probably raised the activity of hydroperoxide lyase (HPL), characterized by low optimal temperature, with the release of a larger number of aldehydes [44]. The concentration of alcohols, ester and ketones did not show any significant differences. However, further investigation is required to better explain the impact of the knife crusher on endogenous enzymes associated with the generation of volatile and phenolic compounds and the relative oxidation process and concerning olive fruits of different genetic origin.

## 4. Conclusions

A rotor in which classic hammers were mounted, commonly used for crushing, and a new model of rotor in which, instead of the hammers, knives are placed radially in relation to the rotor shaft, were used in experimental tests. This study made it possible to understand and hypothesize the prevailing modes of action of the forces applied during olive crushing and explain the implications for quantitative and qualitative aspects observed in the final product. It is the first study that provides an understanding of the forces applied by the crusher tools of the crusher machine: the hammer crusher produces mainly impulsive and compressive stress, while the knife crusher transfers mainly shear stress; both generate the friction phenomena through their impact on the grid and the effects generated on the particle size of the core, the energy transferred to the olive paste and the temperature reached by the pulp in the crushing phase. This information was correlated with results concerning oil quality and the extraction efficiency of the plant. Quality results demonstrated that, when compared to the hammer mill, the new knife rotor used in the crushing phase led to an improvement in VOO quality, mainly in terms of an improvement in volatile and phenolic compounds highly connected to the sensory and health-enhancing properties of the final product. The VOOs extracted using the knife crusher showed a higher concentration of phenolic compounds due to the content of oleuropein and ligstroside derivatives and an increase in the sum of aldehydes mainly responsible for the VOO “green” flavor. No significant difference was found regarding process efficiency, confirming that the ability of the crusher to break the cell walls and vacuoles, thus releasing the oil contained, is comparable using the two different rotors under review.

## Figures and Tables

**Table 1:** Numerical value of crusher tool parameters.

Dimension	Numerical Value [mm]	Crusher
W_H1_	5	HAMMER
W_H2_	15	
W_H3_	20	
H_H1_	25	
H_H2_	20	
H_H3_	15	
R_H_	200	
W_K_	40	KNIFE
H_K_	117	
R_K_	147	

**Table 2:** Comparison of the main parameters of rotor tools.

Parameter	Comparison	Equation
Tool mass (M)	M_H_ = 2.00 M_K_	M_C_ = ρ V_C_
Radius of center of gravity (R)	R_H_ = 1.36 R_K_	-
Moment of inertia (I)	I_H_ = 3.50 I_K_	Equation (6)
Rotational kinetic energy (E)	E_H_ = 3.50 E_K_	Equation (5)
Mass of olives per tool (M)	M_OH_ = 2.76 M_OK_	Equation (7)
Specific energy (e)	e_H_ = 1.27 e_K_	Equation (8)

**Table 3:** Comparison of the main parameters derived from comminution theory.

Parameter	Comparison	Equation
Specific energy	e_H_ = 1.25 e_K_	Equation (10)
Mean temperature of the paste	T_mH_ = 1.25–1.27 T_mK_	Equation (11)

**Table 4:** Quantitative results and process parameters *.

Test Conditions	Maturity Index	POMACE		Extractability
		Moisture (%)	Oil (% db)	(%)
HC	1.6	61.00 ± 1.18 a	5.36 ± 0.54 a	89.79 ± 1.05 a
KC		60.67 ± 1.07 a	5.77 ± 0.18 a	88.86 ± 0.52 a
HC	2.3	64.19 ± 0.64 a	5.81 ± 0.65 a	90.73 ± 0.89 a
KC		64.41 ± 0.99 a	6.39 ± 0.25 a	89.79 ± 0.61 a

* Different letters in column, for each test condition, denote significant statistical differences among means (*p* < 0.05).

**Table 5:** Standard virgin olive oil parameters *.

Test Conditions	Maturity Index	Free Acidity (%)	Peroxide Value (meq O_2_ kg^−1^)	K_232_	K_270_	ΔK
Legal limits for EVOO		≤0.8	≤20	≤2.50	≤0.22	≤0.01
HC	1.6	0.25 ± 0.005 a	3.1 ± 0.5 a	1.672 ± 0.040 a	0.148 ± 0.010 a	−0.028 ± 0.01 a
KC		0.24 ± 0.01 a	3.2 ± 0.1 a	1.675 ± 0.037 a	0.147 ± 0.006 a	−0.009 ± 0.01 a
HC	2.3	0.25 ± 0.00 a	6.0 ± 0.2 a	1.751 ± 0.050 a	0.160 ± 0.010 a	−0.004 ± 0.00 a
KC		0.24 ± 0.02 a	5.1 ± 1.1 a	1.722 ± 0.057 a	0.162 ± 0.003 a	−0.003 ± 0.001 a

* Data are expressed as the mean of three different trials ± standard deviation. Different letters in rows, for each test condition, denote significant statistical differences (*p* < 0.05).

**Table 6:** Phenolic composition of EVOOs. Data expressed as mg kg^−1^ *.

	Maturity Index 1.6		Maturity Index 2.3	
	HC	KC	HC	KC
3.4-DHPEA	5.2 ± 0.5 a	4.3 ± 0.6 b	2.0 ± 0.1 a	1.8 ± 0.2 a
*p*-HPEA	7.5 ± 1.1 a	7.2 ± 0.8 a	2.1 ± 0.1 a	1.8 ± 0.3 a
Vanillic acid	0.3 ± 0.1 a	0.3 ± 0.04 a	0.2 ± 0.03 a	0.2 ± 0.02 a
3.4-DHPEA-EDA	527.1 ± 13.0 b	767.2 ± 12.8 a	484.5 ± 17.6 b	598.2 ± 27.9 a
*p*-HPEA-EDA	225.8 ± 9.0 b	278.8 ± 24.3 a	167.6 ± 8.1 a	170.2 ± 7.0 a
(+)-1-Acetoxypinoresinol	34.5 ± 1.2 b	39.6 ± 2.4 a	28.4 ± 1.0 a	29 ± 1.1 a
(+)-Pinoresinol	21.3 ± 0.6 b	23.8 ± 1.0 a	12.8 ± 0.3 a	12.8 ± 0.5 a
3.4-DHPEA-EA	186.2 ± 10.3 b	246.6 ± 30.5 a	252.6 ± 4.8 b	289.5 ± 13.2 a
Ligstroside aglycone	43.1 ± 2.7 a	44.3 ± 7.2 a	30.2 ± 1.3 a	30.3 ± 1.2 a
Total phenols	1051 ± 21.7 b	1412 ± 51.7 a	980.5 ± 27.6 b	1133.8 ± 45.6 a
Oleuropein derivatives	718.4 ± 16.6 b	1018.1 ± 33.1 a	739.1 ± 18.3 b	889.6 ± 30.9 a
Ligstroside derivatives	276.4 ± 9.4 b	330.2 ± 25.4 a	199.9 ± 8.2 b	202.3 ± 7.1 a
Lignans	55.8 ± 1.3 b	63.3 ± 2.6 a	41.3 ± 1.1 a	41.8 ± 1.2 a

* Data are expressed as the mean of three different trials ± standard deviation. Different letters in rows, for each test condition, denote significant statistical differences (*p* < 0.05).

**Table 7:** Volatile compounds detected in olive oils. Data expressed as µg kg^−1^ *.

	Maturity Index 1.6		Maturity Index 2.3	
	HC	KC	HC	KC
Aldehydes				
Pentanal	n.d.	n.d.	89 ± 9 a	96 ± 2 a
(E)-2-Pentenal	54 ± 4 b	61 ± 2 a	n.d.	n.d.
Hexanal	771 ± 48 a	860 ± 151 a	960 ± 81 a	924 ± 71 a
(E)-2-Hexenal	14292 ± 609 b	19039 ± 1947 a	13267 ± 655 b	16228 ± 1369 a
(E.E)-2.4-Hexadienal	163 ± 7 b	229 ± 42 a	555 ± 34 a	533 ± 39 a
Σ of aldehydes at C_5_ and at C_6_	15280 ± 611 b	20188 ± 1954 a	14870 ± 661 b	17780 ± 1371 a
Alcohols				
Ethanol	n.d.	n.d.	n.d.	n.d.
1-Pentanol	70 ± 10 a	67 ± 5 a	253 ± 26 a	256 ± 22 a
1-Penten-3-ol	379 ± 18a	363 ± 15a	567 ± 83a	244 ± 320a
(E)-2-Penten-1-ol	29 ± 2 b	33 ± 1 a	46 ± 4 a	52 ± 3 a
(Z)-2-Penten-1-ol	299 ± 21 b	352 ± 28 a	793 ± 44 a	791 ± 13 a
1-Hexanol	4361 ± 374 b	4964 ± 291 a	2642 ± 125 a	2725 ± 237 a
(E)-2-Hexen-1-ol	2417 ± 229 a	1931 ± 199 b	1839 ± 101 a	1728 ± 74 a
(Z)-3-Hexen-1-ol	395 ± 8 a	334 ± 27 b	778 ± 16 a	725 ± 51 a
Benzyl alcohol	74 ± 6 a	64 ± 4 b	183 ± 14 a	166 ± 14 a
Phenylethyl Alcohol	238 ± 17 a	243 ± 41 a	621 ± 52 a	657 ± 14 a
Σ of alcohols at C_5_ and at C_6_	8263 ± 439 a	8352 ± 357 a	6919 ± 188 a	6520 ± 410 a
Esters				
Hexyl acetate	29 ± 5 a	33 ± 3 a	34 ± 2 a	38 ± 3 a
(Z)-3-Hexenyl acetate	35 ± 4 a	37 ± 6 a	39 ± 4 a	44 ± 3 a
Σ of esters at C_6_	64 ± 6 a	71 ± 6 a	73 ± 5 a	82 ± 4 a
Ketones				
3-Pentanone	827 ± 57 a	782 ± 81 a	710 ± 18 a	688 ± 56 a
1-Penten-3-one	201 ± 12 a	198 ± 20 a	30 ± 4 a	26 ± 2 a
6-Methyl-5-hepten-2-one	17 ± 2 b	26 ± 3 a	17 ± 1 a	12 ± 1 b
Σ of ketones at C_5_ and at C_8_	1045 ± 67 a	1006 ± 83 a	756 ± 18 a	726 ± 56 a

* Data are expressed as the mean of three different trials ± standard deviation. Different letters in rows, for each test condition, denote significant statistical differences (*p* < 0.05).

## Data Availability

Data are contained within the article.
